# miRNAs regulate acute transcriptional changes in broiler embryos in response to modification of incubation temperature

**DOI:** 10.1038/s41598-018-29316-7

**Published:** 2018-07-27

**Authors:** Watcharapong Naraballobh, Nares Trakooljul, Eduard Murani, Carsten Krischek, Sabine Janisch, Michael Wicke, Siriluck Ponsuksili, Klaus Wimmers

**Affiliations:** 1 Leibniz Institute for Farm Animal Biology, Institute for Genome Biology, 18196 Dummerstorf, Germany; 2 Institute of Food Quality and Food Safety, University of Veterinary Medicine Hannover, D-30173 Hannover, Germany; 3 Department of Animal Science, Quality of Food of Animal Origin, Georg-August-University Goettingen, D-37075 Goettingen, Germany; 4 Faculty of Agricultural and Environmental Sciences, University of Rostock, D-18059 Rostock, Germany

## Abstract

MicroRNAs are post-transcriptional regulators that play critical roles in diverse biological processes. We hypothesize that miRNAs may be involved in regulating transcriptome responses to changes in embryonic incubation temperature in chickens affecting differentiation and proliferation processes during tissue development. Therefore, we conducted comparative transcriptome profiling of miRNAs to examine altered expression in breast and hind muscle of embryos and day 35 chickens experiencing high (38.8 °C), control (37.8 °C), or low (36.8 °C) embryonic incubation temperature during embryonic day (ED) 7–10 or ED10–13. The results revealed differential expression of miRNAs due to modification of embryonic incubation temperature in a muscle type-specific and a developmental stage-specific manner. The immediate effects of thermal change observed in embryos were substantial compared to the subtle long-term effects in chickens at day 35 post-hatch. Upregulation of miR-133 in breast muscle and downregulation of miR-199a-5p, miR-1915, and miR-638 in hind muscle post ED7–10 high-temperature treatment are functionally associated with myogenesis and body size. ED10–13 low-temperature treatment led to downregulation of let-7, miR-93, and miR-130c that are related to proliferation and differentiation. The results provide insight into the dynamics of miRNA expression at variable embryonic incubation temperatures during developmental processes and indicate a major regulatory role of miRNAs in acute responses to modified environmental conditions that affect remodelling of cells and tissues.

## Introduction

Embryonic incubation temperature is a key factor for optimal physiological and developmental processes that may a have long-term influence on adult chickens. Incubation temperature profoundly influences physiological responses via alteration of biochemical reaction rates and protein structures as well as catalytic enzyme functions^1^. Within a limited range, it is critical for broilers to optimize body temperature during pre- and post-hatch processes^2^.

Manipulation of incubation temperature during specific stages of development can result in immediate transcriptomic changes in embryos, although changing temperature beyond critical thresholds can be lethal. Previous studies demonstrated that high temperatures during embryonic day (ED) 7–10 positively associate with improvement of slaughter and breast muscle weights in male broilers, but do not influence meat quality^3^. Our previous experiments showed acute and long-term transcriptomic changes with temperature manipulation during muscle fibre formation. Thermal incubation treatments influence several biological functions and pathways depending on stage of muscle fibre development^4,5^. Hence, manipulation of embryonic incubation temperature may have implications in broiler meat production.

Several studies have indicated that temperature changes impact not only transcriptional changes, but also post-transcriptional regulation in diverse species. In aquatic ectotherms Atlantic cod (*Gadus morhua*), changing incubation temperature during the early somite stage can have significant long-term effects on microRNA (miRNA) activities in juvenile pituitary, gonad, and liver tissues^6^. Another marine species, Senegalese sole (*Solea senegalensis*), induces dynamic expression of several miRNAs during early development at lower incubation temperatures (15 °C)^7^. After entering deep cold torpor, lined ground squirrels (*Ictidomys tridecemlineatus*) and little brown bats (*Myotis lucifugus*) show reduced miR-106b expression, which is associated with lower body temperature during hibernation and is involved in regulation of hypoxia inducible transcription factor-1α (HIF-1α) in skeletal muscle and liver^8^. Further, heat stress alters the expression of several miRNAs in primary cultured human small airway epithelial cells^9^. Altogether, this evidence demonstrates that miRNAs have evolutionarily conserved roles in diverse biological processes, including temperature control.

miRNAs are conserved, non-coding RNAs of approximately 17–22 nucleotides in length that are involved in RNA silencing (cleavage) and post-transcriptional regulation in most, if not all, eukaryotes^10^. Biogenesis of miRNAs involves transcription as long primary transcripts (pri-miRNAs), which are processed to pre-miRNAs and then to mature miRNAs that are ultimately loaded selectively onto the RNA-inducing silencing complex (RISC) to become functional. miRNAs play important roles in numerous biological processes, such as developmental timing, cell death, and cell differentiation, proliferation and transformation. Links between miRNAs and pathological and ontogenetic conditions have been shown in clinical samples^11^. Here we provide evidence for miRNA mediated effects on cell maintenance, differentiation and proliferation in a model using a controlled physical noxae during development. Animal miRNAs partially or perfectly bind target sequences generally at the 3′ untranslated regions (3′-UTR) of target genes^12^. In general, an individual miRNA can regulate hundreds or thousands of target genes, and a single gene can be targeted by several miRNAs. These complex relationships pose a challenge to obtaining discrete results in miRNA studies.

We investigated potential miRNAs involved in regulation of transcriptome responses to modification of embryonic incubation temperature during early (ED7–10) or later (ED10–13) muscle fibre development of broiler-type chickens. In addition to traditional in silico target prediction, we complemented the assignment of miRNA–mRNA relationships and determination of functionally relevant miRNAs derived from this study by using correlation analyses between expression of differentially expressed miRNAs and previously obtained mRNA expression data from the same samples. Observed correlations of miRNA and mRNA from the same samples provide additional experimental evidence for their functional link on top of the in-silico target position. Potential target genes were further assigned for biological functions and pathways.

## Material and Methods

### Design and sample collection

We used hatching eggs from a commercial broiler line (Cobb-Vantress Inc., Siloam Springs, USA) and equally randomly assigned 1,001 hatching eggs to 6 experimental groups. All eggs were incubated in commercial incubators with 12 automatic turns per day (HEKA, Euro-Lux, Riet berg, Germany) at 37.8 °C with 55% relative humidity (RH) until three days prior to hatch. At ED7 all eggs were candled and unfertilized eggs and dead embryos (in total 108 eggs) were removed. During the last days of incubation RH was adjusted to 65% and turning was omitted until hatching (control conditions; continuously for control groups). The experimental thermal profiles comprised of transient adjusting temperature to 38.8 °C (high temperature) or 36.8 °C (low temperature) at early development (ED7–10) or late muscle development (ED10–13), respectively. Accordingly, the experimental design delivered 6 experimental groups in total (38.8 °C, 65% RH, ED7–10 (H10); 2) 37.8 °C, 55% RH, ED7–10 (C10); 3) 36.8 °C, 55% RH, ED7–10 (L10); 4) 38.8 °C, 65% RH, ED10–13 (H13); 5) 37.8 °C, 55% RH, ED10–13 (C13); 6) 36.8 °C, 55% RH, ED10–13 (L13) with 2 repeated batches each. The hatchlings were reared in barn system and fed a standard diet *ad libitum* until day 35 (D35; slaughter) (Supplementary Table S1). Broilers were slaughtered at experimental poultry abattoir of the Department of Animal Sciences (Goettingen, Germany). The processes were proceed by electric stunning (0.12 A, 5 to 10 sec), bleeding, scalded (58 to 60◦C, 3 min) and defeathered. Carcasses were manually eviscerated then dissected and weighted. For each group, breast muscle (*M. pectoralis*) and hind muscle (*M. gastrocnemius*) tissue samples were collected at the respective embryonic stages (ED10 or ED13) and at D35 post-hatch.

Tissue samples were immediately dissected, snap frozen in liquid nitrogen, and stored at −80 °C until use. Zootechnical and biochemical traits were examined as previously described^13^. All animals were sexed, and 6–8 (sex-balanced) animals per experimental group at ED10, ED13, or D35 were used for expression analyses. Study design and sample collection procedures were approved by the Institutional Animal Care and Use Committee (IACUC) of the Department of Animal Sciences of the University of Goettingen, Germany and the Leibniz Institute for Farm Animal Biology and conducted according to the guidelines of the German Law of Animal Protection and the “EU Directive 2010/63/EU for animal experiments”.

### Small RNA isolation

Total RNA was isolated from individual samples (n =144; 6 biological replicates × 6 treatment groups × 2 muscle tissues at ED10 or ED13; 8 biological replicates × 6 treatment groups × 2 muscle tissues at D35) using Tri-Reagent (Sigma-Aldrich, Taufkirchen, Germany), and the small RNA fraction was retained using miReasy and RNeasy MinElute Cleanup kits (Qiagen, Hilden, Germany) with an on-column DNase treatment according to the manufacturer’s protocol. RNA integrity was assessed by capillary electrophoresis using the Agilent Small RNA kit and an Agilent 2100 Bioanalyzer (Agilent Technologies, Waldbronn, Germany). Total RNA concentration was determined using a NanoDrop ND-1000 spectrophotometer (PEQLAB, Erlangen, Germany). Additionally, absence of trace DNA contamination was verified by PCR amplification of *glyceraldehyde 3-phosphate dehydrogenase* (*GAPDH*) in RNA samples. All RNA samples were stored at −80 °C until use.

### microRNA expression

Small RNA fractions (200 ng) were used for sample preparation using a FlashTag BioTin RNA labelling kit (Affymetrix, Santa Clara, CA, USA). Fragmented biotin-labelled cRNAs were further hybridized for 16 hours to an Affymetrix GeneChip miRNA 3.0 Array containing 19,724 probe-sets designed from 153 species based on miRBase version 17. After staining and washing on an Affymetrix Fluidics Station 450, arrays were scanned on an Affymetrix G3000 Gene Array Scanner. Raw data were pre-processed using Affymetrix GCOS 1.1.1 software.

### Data processing and statistical analysis

Raw probe signal intensity was pre-processed and normalized using Perfect Matched and Detection Above Background features of Affymetrix Expression Consol software. Data were submitted to the MIAME-compliant database Gene Expression Omnibus (accession number: GSE83703-GSE83704) accessible via the National Center for Biotechnology Information (www.ncbi.nlm.nih.gov/geo). Differential expression of miRNAs was computed by analysis of variance (JMP Genomics, SAS-Institute, Cary, NC, USA). Independent calculation was performed for each tissue. Fixed effects of temperature, treatment period, and their interactions were modelled in statistical tests. For analysis in D35 samples, slaughter weight was used as covariance in the statistical model. Differentially expressed miRNAs were identified by comparing treatment groups and corresponding controls for ED7–10 or ED10–13. Significance threshold was set at nominal p ≤ 0.05. To account for multiple testing, FDR values were calculated based on the p-value distribution using an approach designed for large data sets^14^.

### Functional miRNAs and potential target genes

**Figure 1:**
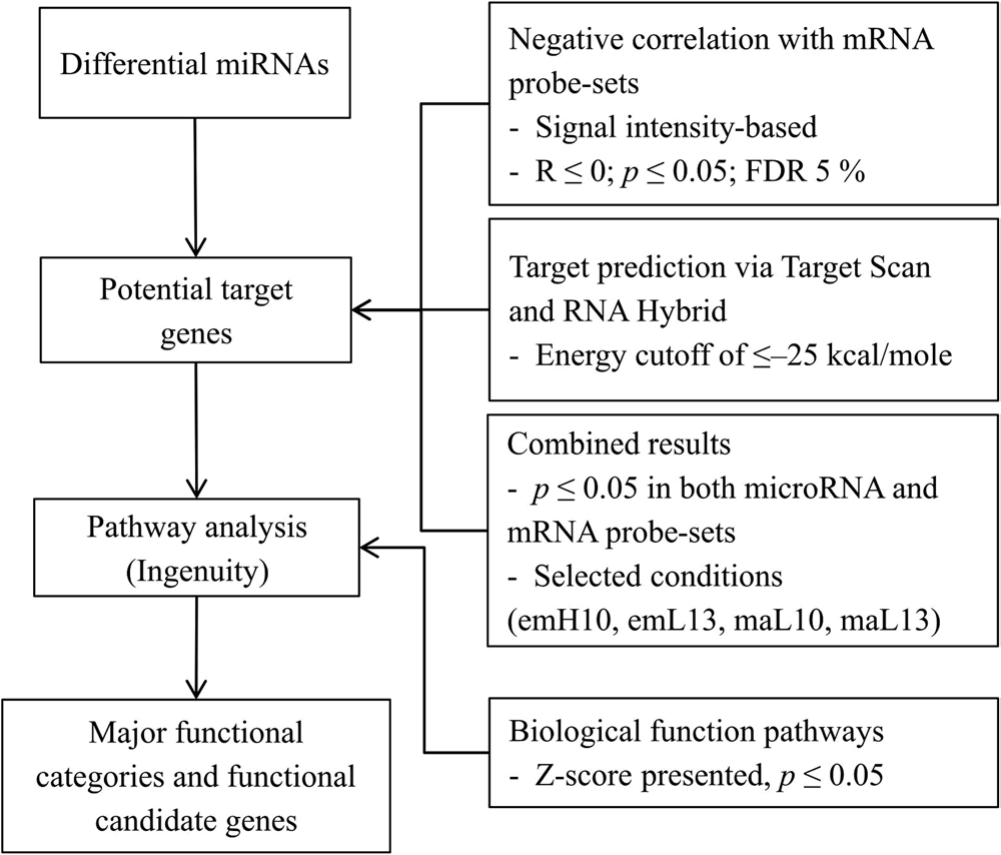
microRNA functional analysis pipeline.

To identify functional miRNAs and potential target genes, we integrated miRNA expression data from the present study and mRNA expression data of samples from our previous publications^4,5^ using correlation analysis and target prediction (Fig. 1). Firstly, correlation analyses between signal intensities of differentially expressed miRNAs and all mRNA probe-sets were calculated. All significant negative correlations between miRNA–mRNA pairs were retained (*p* ≤ 0.05; 5% FDR). Secondly, all differentially expressed miRNAs were scanned for potential target genes against all available chicken mRNA sequences in the NCBI database using Target Scan software^15^. Predicted targets were further filtered using RNA Hybrid software^16^ with an energy threshold cut-off of ≤ –25 kcal/mole. Accordingly, a “functional” miRNA was defined as a differentially expressed miRNA that negatively correlated with mRNA transcriptional level and was predicted to bind at the respective target genes.

### Pathway analysis

Differentially expressed miRNAs and their potential targets were mined for biological functions and gene regulatory networks using Ingenuity Pathway Analysis (QIAGEN Inc., https://www.qiagenbioinformatics.com/products/ingenuity-pathway-analysis). Statistical significance was determined based on Fisher’s exact test (*p* ≤ 0.05). Significant biological pathways for each list of miRNAs and target genes were aggregated into new major categories based on shared GO subterms to simplify results, while maintaining a comprehensive view of biological processes.

Eight major biological functional groups were defined: (1) cell maintenance, proliferation, differentiation, and replacement; (2) organismal, organ, and tissue development; (3) nutrient metabolism; (4) genetic information and nucleic acid processing; (5) molecular transport; (6) cell signalling and interaction; (7) small molecule biochemistry; and (8) response to stimuli. Significant pathways were further considered “activated” or “deactivated” based on positive or negative Ingenuity z-scores that predict the direction of regulation of a pathway. Selected genes were used to generate regulatory networks based on best z-scores, *p*-values, and biological functions related to tissue development and myogenesis.

### Quantitative real time PCR (qPCR) validation

Six miRNAs (gga-miR-199, gga-miR-30d, gga-miR-460a, gga-miR-100, gga-miR-222, gga-miR-133a) differentially expressed in embryo breast were validated by qPCR of the same individual samples that were used for miRNA-chips (n = 32). The cDNA was synthesized from 250 ng isolated miRNAs using Mir-X™ miRNA First-Strand Synthesis and SYBR® qRT-PCR Kit (Clontech Laboratories, Mountain View, USA) according to manufacturer’s protocols. All measurements were performed in duplicates. The thermal parameters were 50 °C for 2 min, 95 °C for 2 min, followed by 45 cycles of 95 °C for 15 sec and 60 °C for 1 min. The universal qPCR primer was provided in the kit and the miRNA-specific forward primers were designed for the miRNAs (Supplementary Table S2). Internal standard of Ce_miR-39_1 miScript Primer Assay (QIAGEN, Germany) was used for normalize the miRNA expression value. The designed primer sequence information is accessible in Additional file. Correlation coefficient analysis between the miChip and qPCR data was calculated using SAS 9.3 (SAS Institute).

## Results

### Differentially expressed miRNAs

Although the Affymetrix GeneChip miRNA 3.0 array contains multiple mature miRNAs from diverse species that may resemble a chicken miRNA family, we treated each mature miRNA probe-set as an entity (feature) in statistical tests and then aggregated significant probe-sets into unique mature miRNAs. The number of differential probe-sets and mature miRNAs (unique miRNAs) for each tissue type, temperature treatment condition, and sampling stage are summarized in Table 1.

**Table 1:** Differentially expressed miRNAs (*p* ≤ 0.05).

	Treatment (ΔC)	Total^c^ miRNAs (breast)	Unique^c^ miRNAs (breast)	Regulation		Total^c^ miRNAs (hind)	Unique^d^ miRNAs (hind)	Regulation	
				Up	Down			Up	Down
Embryo^a^	H10–C10	694	243	158	85	603	262	174	88
	H13–C13	380	160	71	88	362	211	123	88
	L10–C10	455	201	113	88	536	200	60	140
	L13–C13	733	316	204	112	401	224	90	134
D35^b^	H10–C10	148	107	74	33	98	88	31	57
	H13–C13	380	165	114	51	1328	419	176	243
	L10–C10	154	80	48	32	881	419	146	273
	L13–C13	165	85	69	16	1808	550	146	404

^a^significance threshold nominal *p* ≤ 0.05 corresponding to FDR-adjusted *p* ≤ 0.18.

^b^significance threshold nominal *p* ≤ 0.05 corresponding to FDR-adjusted *p* ≤ 0.18 or *p* ≤ 0.71 in hind and breast muscle, respectively.

^c^Number of probe-sets on the microarray (redundantly counting the same kind of miRNA from different species).

^d^Each kind of miRNA (unique sequences only counted once).

Numerous differentially expressed mature miRNAs were detected in all comparisons. In analyses comprising breast muscle (embryonic stage) and hind muscle (embryonic stage, adult stage) the threshold level for nominal p-values corresponded to FDR values of 0.18; for analyses comprising the breast muscle at adult stage, nominal p-values corresponded to a FDR value of 0.71 (Table 1). Quantitative real time PCR of exemplarily selected miRNAs in breast muscle revealed significant correlations with microarray data (Supplementary Figure S1). The lists of miRNAs differentially expressed across comparisons of temperature treatments, tissue types, and sampling stages, with a comparable number of upregulated and downregulated miRNAs, offer a rich source for subsequent filtering based on the biological context and function-related criteria. The results suggest that volatile miRNA changes in terms of direction of regulation and timing regulate transcriptional alterations due to modification of embryonic incubation temperature.

### Functional miRNAs and potential target genes

Integrating miRNA expression data with previous gene expression profiles of matched samples (www.ncbi.nlm.nih.gov/geo; accession number: GSE76670) using correlation analysis revealed “functional” miRNAs with negatively correlated miRNA–mRNA relationships. From our previous study, we found that increasing temperature from 37.8 °C to 38.8 °C during ED7–10 (H10) and decreasing temperature from 37.8 °C to 36.8 °C during ED10–13 (L13) resulted in considerable immediate transcriptomic changes (based on the abundance of differentially expressed genes) in embryos, whereas decreasing temperature during ED7–10 (L10) as well as ED10–13 (L13) showed large long-term effects in D35 chickens^4,5^. Therefore, we focused on these treatment conditions that showed most prominent changes of the mRNA expression.

Numbers of miRNA–mRNA pairs, covering potential miRNA-targeted genes negatively correlated with miRNAs and also predicted as miRNA binding sites, and unique miRNAs are presented in Table 2. Further information on criteria and pipelines are shown in Fig. 1. Overall, embryos had higher numbers of potential miRNA–mRNA relationships, ranging from 104 miRNA–mRNA pairs for L13 in hind muscle to 941 pairs for H10 in breast muscle, compared to D35 chickens (2 pairs for L13 in hind muscle and 19 pairs for L10 in breast muscle).

**Table 2:** Functional miRNAs and potential target genes for selected treatment conditions.

Stage	Treatment Tissue		miRNA–mRNA pairs	mRNA targets	Unique miRNAs
Embryo	H10–C10	Breast	941	421	40
		Hind	444	200	38
	L13–C13	Breast	394	168	50
		Hind	104	49	25
D35	L10–C10	Breast	19	10	1
		Hind	10	8	7
	L13–C13	Breast	7	6	5
		Hind	2	1	2

For H10, 421 unique genes were negatively correlated with 40 miRNAs and 200 unique genes were predicted as target candidates for 38 miRNAs in breast and hind muscles, respectively. Number of miRNA–mRNA pairs in breast muscle was higher than in hind muscle at *in-ovo* stages. A similar number of miRNAs and potential targets were also identified in L13 in both muscle types. Only up to 10 miRNA–mRNA pairs were found among treatment conditions and muscle types at D35 (Table 2).

**Figure 2:**
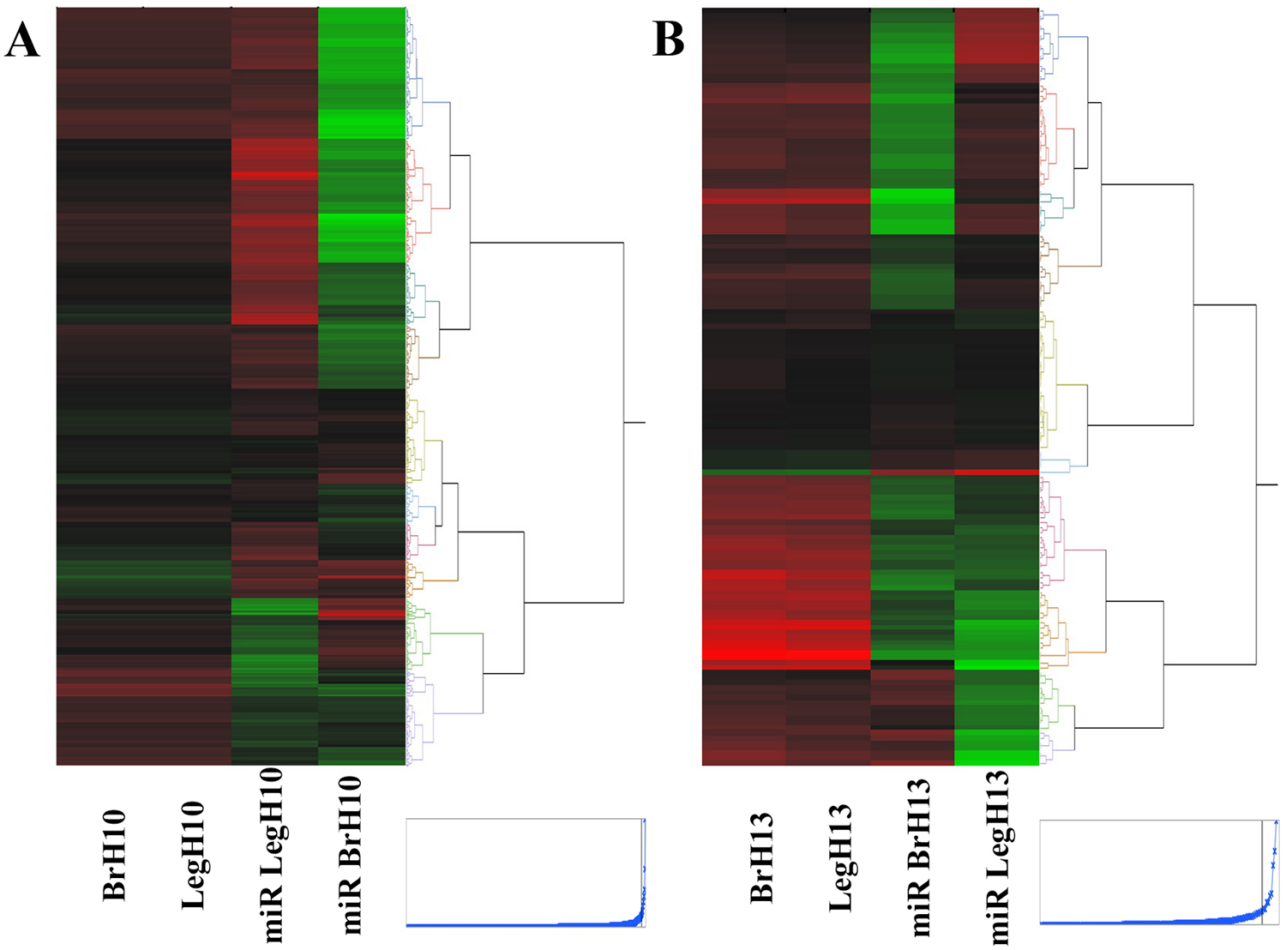
Expression-based (least-squares means) hierarchical clustering of differential miRNAs and potential mRNA targets derived from embryonic breast and hind muscle after H10 (**A**) or L13 (**B**) treatment.

Differential miRNAs and potential target genes were further used to generate a hierarchical clustering based on expression level to demonstrate an overall dominantly negative correlation between miRNA and mRNA expression levels (Fig. 2). Altogether, these results suggest that miRNAs may play an essential regulatory role on the immediate transcriptome response to modification of incubation temperature.

### Pathway analysis

To functionally link miRNAs to the physiological effects of modifications of embryonic incubation temperatures, differentially expressed miRNAs and potential target genes were analysed using Ingenuity and its Knowledge Base software. To simplify and comprehend the resulting complex biological pathways and key genes, we aggregated significant terms and pathways into “major functional categories” based on shared terms and keywords of those pathways. Pathways in significant functional categories for each treatment condition at embryonic stages are given in Supplementary Tables S3 and S4.

In breast muscle of embryos, H10 showed activation of pathways related to cell maintenance and proliferation, organismal and tissue development, and nutrient metabolism, while L13 showed stimulated cell maintenance and proliferation, organismal and tissue development, genetic information and nucleic acid processing, cell signalling, and interaction and response to stimuli. Hind muscle of embryos showed deactivation of cell maintenance and proliferation, while L13 affected cell maintenance, proliferation, and differentiation pathways. Detailed information of pathway analyses can be found in Supplementary Tables S3 and S4. Functional miRNAs obtained from H10 and L13 that were highly associated with various target genes and that therefore were related to at least 4 out of 8 major categories are provided in Tables 3 and 4.

**Table 3:** Differential miRNAs targeting genes in significant pathways in embryonic breast and hind muscles affected by H10 treatment.

Target	miRNA	*p*-value	FDR	Fold change (ΔC)	Regulation
Breast	miR-133	0.0484	0.1691	1.56	Up
	miR-1825	0.0362	0.1386	1.84	Down
	miR-199a-3p	0.0002	0.0023	1.47	Down
	miR-212-star	0.0155	0.0750	2.27	Up
	miR-222	0.0355	0.1366	1.41	Up
	miR-289	0.0137	0.0684	1.63	Up
	miR-4530	0.0476	0.1671	1.73	Up
	miR-460-5p	0.0046	0.0302	1.85	Down
	miR-5109	0.0364	0.1389	1.34	Up
Hind	miR-1915	0.0262	0.1223	1.63	Down
	miR-199a-5p	0.0368	0.1533	1.38	Down
	miR-212	0.0138	0.0786	2.34	Up
	miR-2861	0.0306	0.1359	1.68	Down
	miR-3885-5p	0.0119	0.0707	1.72	Down
	miR-3960	0.0133	0.0763	1.59	Down
	miR-4454	0.0044	0.0350	1.61	Down
	miR-4592	0.0003	0.0040	2.30	Down
	miR-638	0.0054	0.0403	1.67	Down

**Table 4:** Differential miRNAs targeting genes in significant pathways in embryonic breast and hind muscles affected by L13 treatment.

Target	miRNA	*p*-value	FDR	Fold change (ΔC)	Regulation
Breast	let-7	0.0130	0.0658	1.85	Down
	miR-130c	0.00001	0.0012	1.54	Up
	miR-1677	0.0004	0.0048	2.01	Up
	miR-17-3p	0.0158	0.0761	1.56	Up
	miR-1908	0.0217	0.0962	1.86	Down
	miR-199b	0.0004	0.0044	1.87	Down
	miR-222	0.0017	0.0139	1.72	Up
	miR-312-5p	0.0480	0.1680	1.76	Up
	miR-460-5p	0.0267	0.1117	1.59	Down
	miR-4651	0.0198	0.0899	1.72	Down
	miR-4900a	0.0058	0.0361	2.30	Down
	miR-5109	0.0044	0.0292	1.51	Down
	miR-762	0.0329	0.1296	1.57	Down
	miR-92a	0.0015	0.0127	2.95	Up
	miR-92b	0.0463	0.1638	1.59	Down
	miR-93	0.0176	0.0822	1.55	Down
	miR-138	0.0480	0.1815	1.39	Up
Hind	miR-2137	0.0047	0.0363	1.99	Down
	miR-222	0.0359	0.1511	1.44	Up
	miR-271	0.0034	0.0283	2.74	Down
	miR-383	0.0010	0.0113	3.58	Up

### Regulatory networks predicting potential physiological effects

**Figure 3:**
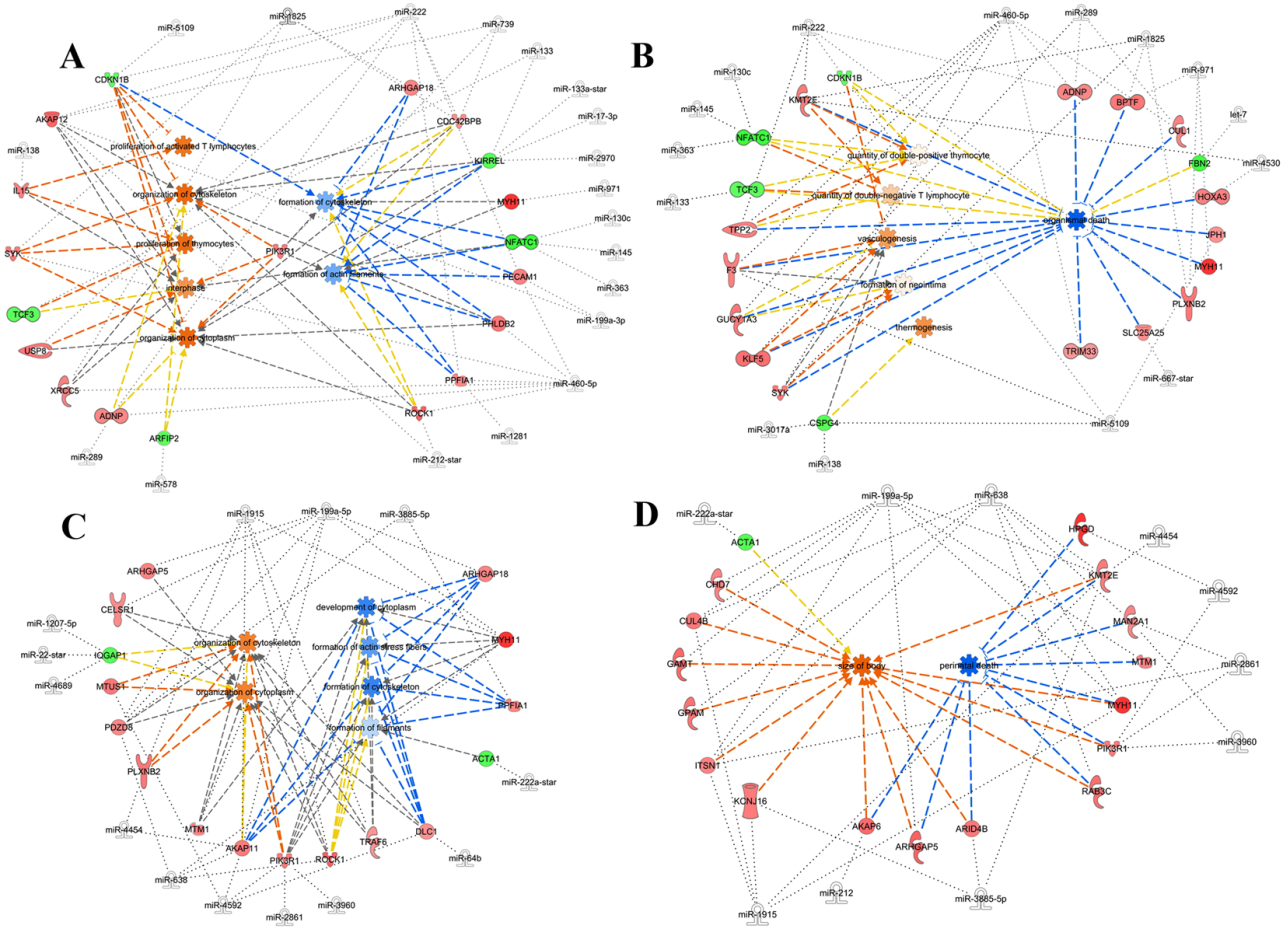
miRNA–mRNA regulatory networks. Representative gene regulatory networks derived from breast muscle (**A** and **B**) or hind muscle (**C** and **D**) of H10 group that are related to functional category group 1 (maintenance, proliferation, differentiation, and replacement of cells) (**A** and **C**) or group 2 (organ and tissue development) (**B** and **D**). Activated pathways are orange, while deactivated pathways are blue. The networks were generated through the use of IPA (QIAGEN Inc., https://www.qiagenbio-informatics.com/products/ingenuity-pathway-analysis).

**Figure 4:**
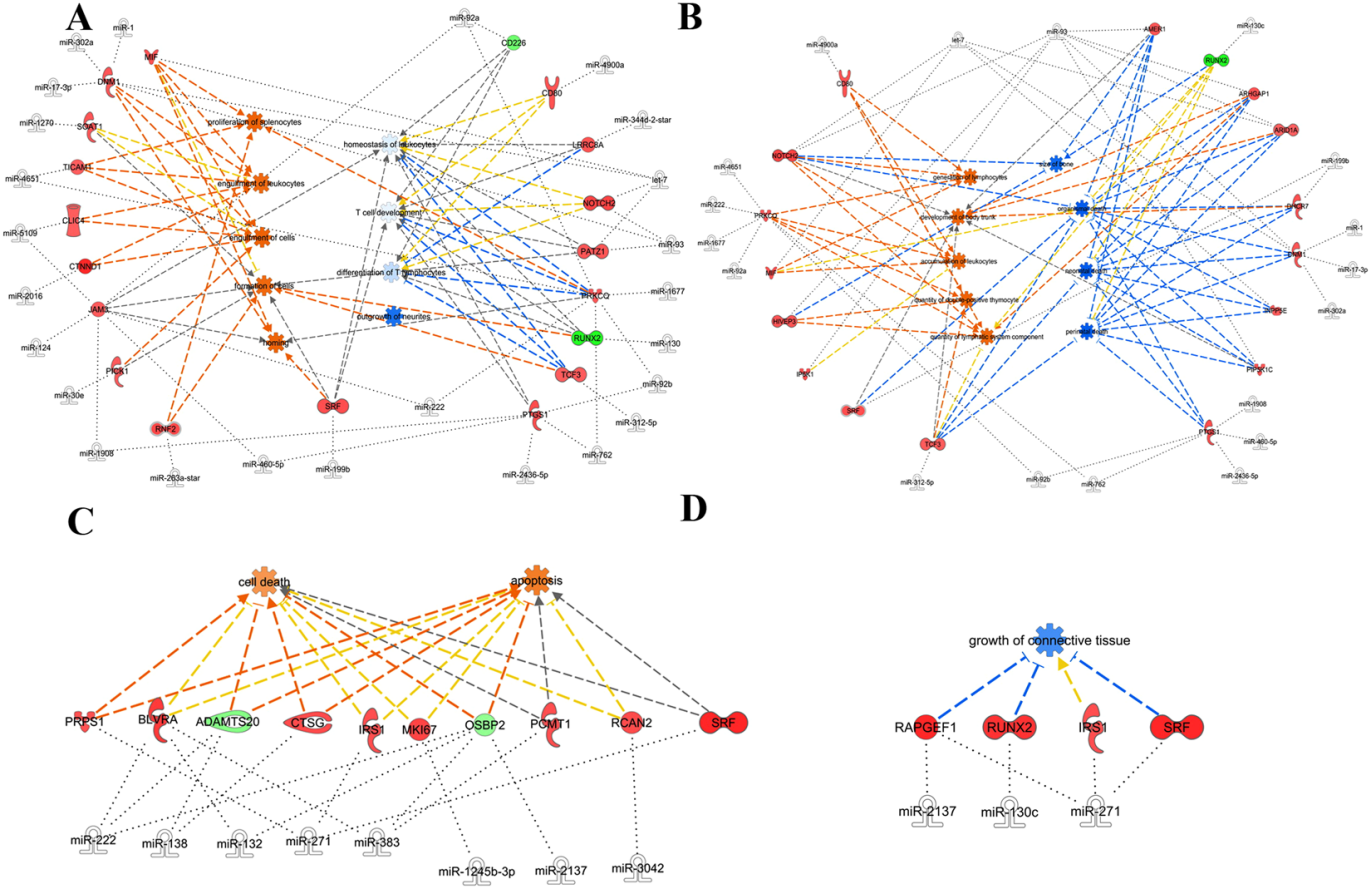
miRNA–mRNA regulatory networks. Representative gene regulatory networks derived from breast muscle (**A** and **B**) or hind muscle (C and **D**) of L13 group that are related to functional category group 1 (maintenance, proliferation, differentiation, and replacement of cells) (**A** and **C**) or group 2 (organ and tissue development) (**B** and **D**). Activated pathways are orange, while deactivated pathways are blue. The networks were generated through the use of IPA (QIAGEN Inc., https://www.qiagenbio-informatics.com/products/ingenuity-pathway-analysis).

Representative miRNA–mRNA regulatory networks were modelled for H10 (Fig. 3) and L13 (Fig. 4) treatment in breast and hind muscles. The networks integrate miRNAs, potential target genes, and Ingenuity biofunctions and display an enrichment of miRNA–mRNA pairs related to major functional categories of cell maintenance, proliferation, and differentiation as well as tissue and organ development (Supplementary Tables S3 and S4).

Representative miRNA–mRNA regulatory networks demonstrate complex connectivity and relationships between the two molecular features. Sets of miRNAs target several genes that assemble into biological pathways and hence regulate these pathways. For example, miRNAs derived from H10 revealed activation of cytoskeletal organization and inhibition of cytoskeletal formation, demonstrating the overall fine tuning and balancing impact of miRNA posttranscriptional regulation (Fig. 3A,C). We also observed activation of pathways involved in white blood cell quantity, vasculogenesis, and thermogenesis as well as stimulation of body size. Pathways involved in reduced organismal death and perinatal death are shown in Fig. 3B,D.

Breast muscle that experienced reduced incubation temperature during ED10–13 showed stimulation of pathways related to proliferation, activity, formation, differentiation, and homeostasis of white blood cells. Regulated pathways included reduced growth of neurites (Fig. 4A). In hind muscle, we identified pathways involved in activation of apoptosis and cell survival (Fig. 4C). In breast muscle, we identified pathways related to white blood cell quality and development of body trunk. We also identified deactivation of organismal death and bone size (Fig. 4B) and, in hind muscle, inhibition of growth of connective tissue (Fig. 4D). Additional information for all pathways derived from miRNA–mRNA relationships indicated from integrated data analysis is available in Supplementary Figs S2–S5.

## Discussion

Accumulating evidence suggests that modification of embryonic incubation temperature can result in phenotype variations of D35 chickens, such as adaptation to environmental conditions like heat stress. We have previously reported that changing incubation temperature during embryonic myogenesis influences weight gain and meat quality of broilers^13^. High temperature (38.8 °C) between ED 7 to 10 or ED 10 to 13 was found to be associated with higher body weights of D35 broilers compared to broilers from complementary normal (37.8 °C) or lower (36.8 °C) temperature groups^13^. Late increase of body weight at adult stage was shown to be due to increased size of breast muscle rather than hind muscle^3^.In fact, whereas breast muscle showed a 5.7% increased weight after high temperature compared to the control (351 g vs. 331 g) leg muscle showed only 3.9% increase (381 g vs. 367 g). This is in line with the fact that the treatments coincide with secondary fibre development and thus may have stronger impact on the breast muscle dominated by type II fibres originated from secondary fibres than on the hind muscle mainly consisting of type I fibres originated from earlier developing primary fibres. Moreover, lowered incubation temperature had in immediate effect on the *in-ovo* body weight that was reduced at the respective time points ED10 and ED13; higher incubation temperature slightly but non-significantly increased the embryo weight^17^. Also the D35 animals used in this study had higher body weight after transiently incubated at higher temperature between ED7–10 or ED10–13, whereas lower incubation temperature showed no effect on body weight later in life.

Further, we have previously demonstrated that both increasing and decreasing incubation temperature (1 °C from the control, 37.8 °C) immediately affects transcriptome profiles of embryonic muscle and associates with transcriptional changes of muscle of D35 chickens, indicating potential long-term transcriptomic effects of treatment during embryonic incubation temperature to adult stage^4^. This study now establishes posttranscriptional regulation by miRNAs in the above phenomenon.

Indeed, we found many differentially expressed miRNAs after thermal incubation treatments at the embryonic stage, compared to only a few differential miRNAs in D35 chickens. These results suggest that miRNAs are primarily, immediately affected and contribute to the regulation of gene expression at *in-ovo* stages in acute response to environmental circumstances during embryonic development, when thermoregulatory systems are not yet fully functional. In contrast there are only subtle changes of miRNAs in D35 chickens associated with embryonic thermal treatment. Mechanisms other than miRNA regulatory networks seem to be more important for long-term transcriptional changes in response to *in-ovo* thermal treatment such as epigenetic modifications which might have more persistent effects on mRNA expression.

For functional analysis, we focused on those thermal treatment conditions ((H10) and (L13) in embryos (L10) and (L13) in D35 chickens) that were previously found to show the most prominent mRNA-transcript changes^4,5^. Pairs of miRNAs and mRNAs were identified by in-silico target prediction complemented by experimental evidence based on the correlation analysis of miRNA and mRNA expression data obtained from identical samples. Considerable numbers of miRNAs and potential mRNA targets that were both regulated in the context of the experimental conditions were detected for H10 (high temperature during ED7–10) and L13 (low temperature during ED10–13) for *in-ovo* stages. Biological functions of the identified differential miRNAs at H10 and L13 in the regulatory contexts with their potential target genes were considered using Ingenuity analysis software and Knowledge Base.

### H10: High temperature during ED7–10

Within each major category of functions, networks linking target mRNAs, miRNAs, and biofunctions display the multi-connectivity of these elements (Figs 3 and 4 for major categories 1 and 2; Supplementary Figure S2–S5 for remaining major categories). While most knowledge about the functional role of miRNAs comes from studies of pathological conditions, in particular cancer, our results provide evidence for miRNAs’ role in ontogenetic proliferation and differentiation processes. In fact, H10 treatment consistently shifts expression of miRNAs related to these cellular developmental processes at *in-ovo* stages. Moreover, H10 treatment promotes pathways related to organismal survival and carbohydrate metabolism. In particular, thermogenesis, which is involved in thermoregulation, is important in breast muscle but not hind muscle. At the level of biofunctions, L13 treatment affects pathways related to cellular and organismal development via processes of proliferation, differentiation, and death. This is similar to H10 conditions; however, largely different miRNAs and target genes are shifted, indicating that alternative pathways are addressed to keep conditions close to homeostasis.

In breast muscle, two differential miRNAs, miR-138 (Supplementary Tables S3 and S4) and miR-3017a (Supplementary Tables S3), relate to genes that were enriched for major category 2 and predicted for activating thermogenesis at H10. Among those target genes was *CSPG4*, which is known to be involved in vasculogenesis. Interestingly, 12 miRNAs, including miR-133, miR-199, and miR-212, were associated with genes in major category 3 that are related to metabolism and synthesis of carbohydrates: upregulated *ADRB2*, *CX3CL1*, and *PPP1R3B*; and downregulated *CHPF* and *CHST3*. While *PPP1R3B* was found in liver and skeletal muscle tissues, it is also involved in regulating glycogen synthesis by forming a glycogen-targeting subunit for phosphatase PP1^18^.

In hind muscle at H10 conditions, differentially expressed miRNAs, including miR-199a-5p, miR-1915, and miR-638, were related to 14 genes in major category 2, which is associated with accumulated body size and reduced perinatal death, including *CUL4B*, *ITSN1*, *MLL5*, and *MYH11*. Interestingly there is evidence that miR-1915 is involved in apoptosis via interaction with the antiapoptotic protein Bcl-2 and that miR-638 relates to vascularization^19,20^. In fact, pathways analysis here indicates corresponding links to perinatal death and cellular development (Fig. 3). Further, the results show that hydrolysis of carbohydrates mediated by genes such as *MTM1*, *NT5E*, *PLCB1*, and *MGLL*, which are related to nutrition metabolism in major category 3, are targeted by miRNAs shifted at H10 in hind muscle (miR-199a-5p, miR-212, and miR-222).

Interestingly, both tissues of the H10 group showed stimulation of major category 1, organization of cytoskeleton and cytoplasm, which is linked to several miRNA-targeted genes, including *CDKN1B*, *MTUS1*, *PIK3R1*, *PLXNB2*, and *SYK*. Especially *PIK3R1*, which is represented in multiple functional categories, was predicted to be a potential target of miR-739 in breast muscle and miR-2861, miR-3960, and miR-4592 in hind muscle. In addition, several target genes were associated with improved survival in major category 2 by deactivating organismal death and perinatal death in both breast and hind muscle.

Among the differentially expressed miRNAs miR-133 is prominent as it is known as a muscle-specific miRNA also called “myomiR,” affecting muscle proliferation, myotube formation, and differentiation^21,22^. Chen *et al*. (2006) showed that upregulation of miR-133 was associated with myoblast proliferation but reduced cell differentiation^23^. In chickens, miR-133a was shown as stimulatory factor in late-stage development in response to myogenin^24^. In this study miR-133 was upregulated in breast muscle but not in hind muscle. This is in line with the differential expression of miR-133 found in porcine M. longissimus dorsi and M. psoas major, i.e. white and red muscles with different proportions of white (type II) and red (type I) muscle fibres like breast and hind muscle^20^. Moreover, differential expression of miR-133 in the two muscles may also be due to different degree of proliferation and differentiation processes in the muscles reflecting the subsequent development of white fibres (FTG) in breast muscle and red fibres (STO) in hind muscle.

MiR-212 revealed pleiotropic properties in various tissues so far mostly in relation to pathological states like cancer and inflammation and mostly related to neuronal cells^25^. We previously found miR-212 among co-expressed miRNAs that affect muscle properties related to meat quality in pigs^26^. Here miR-212 showed increased abundance in breast and hind muscle at H10 where it was assigned functional roles in several pathways related to cellular and organismal growth (Fig. 3).

Overall, H10 treatment effects tended to promote high body and muscle weight compared to low temperature treatment, which is in line with previous observations^17^. Accordingly, shifts of miRNA expression mainly affect pathways related to cell survival, organization of cytoskeleton and cytoplasma, angiogenesis and vascularization and also more specific pathways of myogenesis.

### L13: Low temperature during ED10–13

Compared to H10 treatment, L13 had less differentially expressed genes. Low temperature treatment during ED10–13 (i.e., formation of secondary fibres during myogenesis) activated several miRNAs that in turn regulate major categories 1 and 2. In breast muscle, major category 1 showed potential activation of 23 biological functions, such as cellular activity of formation and engulfment. Formation of cells was influenced by upregulation of *RNF2* and *TCF3* and downregulation of *RUNX2*. These genes were targeted by miR-130c, miR-263a-star, and miR-312–5p. Moreover, other differentially expressed genes, including *JAM3*, *PATZ1*, *PICK1*, *SOAT1*, and *SRF*, were also associated with these miRNAs.

It is interesting that differential miRNAs from L13 treatment related to the predicted tendency of reduced bone size. Target genes *NOTCH2*, *HIVEP3*, *AMER1*, and *RUNX2* were regulated by downregulation of let-7, miR-93, and miR-130c. L13 treatment is associated with low body weight of embryos compared to the high temperature^17^. Muscle size is a major force inducing adaptation of bone size. It is speculated that reduced bone size could be well correlated and of consequence of low body weight.

Furthermore in breast muscle at L13 eight differentially expressed genes and 16 differential miRNAs, including let-7, miR-92, and miR-93, belonged to major category 3, with biofunctions of uptake of carbohydrate and D-glucose.

Let-7 has been a major topic of discussion for functional roles of miRNAs. miRNAs of the let-7 family play major roles in regulating proliferation during ontogenesis and particularly during myogenesis^22^. Let-7 family members are associated with aging in humans and downregulation of cell cycle control, such as cellular proliferation and differentiation pathways^27^. Moreover, during myogenesis, let-7 can suppress Dicer and HMGA2, which have roles in adipogenesis and mesenchymal differentiation^28^. Interestingly, long non-coding RNA H19, possessing multiple let-7 binding sites, is proposed to prohibit let-7 from binding to other targets^29^. Here let-7 showed different abundance due to L13 treatment in breast muscle and was assigned to various pathways related to cellular, organismal and metabolic processes. Let-7 was not found differentially expressed in hind muscle at L13.

miR-93 can potentially downregulate *AKT3*, which reduces proliferation and facilitates differentiation of myoblasts in skeletal muscle development^30^.

miR-130c is related to thermal regulation in various species, especially in aquatic ectotherms. Previous studies showed that Atlantic cod (*Gadus morhua*) have less miR-130c transcripts during early somite formation at 9.5 °C incubation temperature^6^. Another marine species, Senegalese sole (*Solea senegalensis*) vigorously expresses several miRNAs, including miR-130c, during early development (20-somite stage) at lower incubation temperature (15 °C)^7^.

For hind muscle, major category 1 relates to initiation of cell death and apoptosis via multiple differentially expressed genes, including downregulated *ADAMTS20* and *OSBP2* and upregulated *PRPS1*, *RCAN2*, and *SRF*. Multiple miRNAs, including miR-132, miR-138, miR-222, miR-271, miR-383, miR-1245b-3p, miR-2137, and miR-3042, are well correlated with this functional category. *IRS1*, *RAPGEF1*, *RUNX2*, and *SRF* are targeted by miR-130c, miR-271, and miR-2137. Further, an activated biological function in major category 8 was cell movement of neutrophils, which relates to *CTSG*, *PRKCQ*, *SRF*, and *TSC1*. These genes are targeted by miR-138, miR-222, miR-271, and miR-383.

We reported that L13 treatment is associated with low body weight of embryos compared to high temperature treatment^17^. Bone and muscle are highly associated during developmental stages^31^, so reducing bone size could also correlate with low body weight *in-ovo*.

### miRNAs shifted at H10 and L13

Due to their functional annotation to several biofunctions and functional networks and significant response to both treatments and/or in both muscles a number of miRNAs, including members of the miR-199 family, miR-222, miR-460, and miR-5109, play important roles in fine tuning gene expression at the background of various mRNA expression profiles given in breast and hind muscle at day 10 and day 13 of *in-ovo* development.

Members of the miR-199 family are involved in multiple roles, including stem cell differentiation and embryo development^32^. As reviewed by Gu and Chan (2012), miR-199 family is differentially expressed in various tissues and pathological states as well as developmental processed, providing an example of the versatile function and regulation of miRNAs^32^. Accordingly, in our study miR-199 family members showed different abundances in breast and hind muscle at H10 and in breast muscle at L13 and were assigned to developmental processes at the cellular and organismal levels but also to metabolic processes as shown in Figs 3 and 4 and supplemental materials.

Due to its impact on cell proliferation and apoptosis in cancer, functions in physiological and pathological processes in the cardiovascular system and responsiveness to environmental exposure miR-222 has been proposed as a biomarker for cancer, cardiovascular diseases and environmental stressors^33–35^. Here we found regulation of miR-222 breast and hind muscle at H10 and L13 conditions, thus in samples where proliferative process are important, i.e. at early *in-ovo* development, and that were exposed to environmental stressor, albeit physical stress not chemical stressor as in studies reviewed by Vrijems *et al*. (2015). Interestingly, miR-222 mediates atrophy in denervated fast muscles in rates, i.e. processes that involve rearrangement of fast and slow muscle fibres as during myogenesis that is addressed in our study^36^.

For miR-5109 and miR-460 that were both differentially expressed in breast muscle at H10 and L13 knowledge of their function is scarce. MiR-460 showed lowered abundance in breast muscle at H10 and L13. Interestingly, miR-460 was detected as a growth related miRNA in tilapia^37^. Our study of functional mi-RNAs with their linked mRNAs revealed that miR-460 is involved in many structural and metabolic cellular processes but not directly linked body growth. MiR-5109 showed increased abundance at H10 but decrease at L13. There is no further information about miR-5109 available. The divergent regulation at H10 and L13 might be due to the direction of temperature change or due to different developmental stages that both are associated with specific mRNA expression profiles.

For miR-199, miR-222, miR-460 we found the same direction of regulation in breast and hind muscle and/or at H10 or L13 conditions. This reflects the complexity of miRNA expression and its role for regulation of mRNA transcript abundance; where due to the numerous potential target genes of each single miRNA changes of their frequency contribute to the maintenance of homeostasis at the background of different mRNA profiles. MiRNAs can be assigned to functional pathways via their link to target genes. Therefore, in order to get an comprehensive knowledge, global studies of expression profiles at various biology-based systems are required^32^. Our study contributes to this collection of knowledge and particularly provides *in-vivo* data from a non-pathological organism at different developmental stages.

### Summary

In the present study, we have demonstrated that modification of embryonic incubation temperature immediately affects miRNA expression profiles of breast and hind muscles of chicken embryos and is associated with altered expression of miRNAs in D35 chickens. An integration analysis of miRNA data and previous matched-sample mRNA data revealed functional miRNAs and enabled assembly of miRNA–mRNA regulatory networks related to biological pathways and potential physiological effects, though the functionality and targeting of each single miRNA and corresponding mRNAs remains to be validate by adequate *in-vitro* studies

Differentially expressed miRNAs and targeted mRNAs showed treatment condition specificity in terms of timing of treatment (ED7–10 or ED10–13), tissue type, and stage of development. The large repertoire of miRNA–mRNA pairs that are shifted in various experimental groups but that finally fine-tune similar biofunctions reflects a considerable functional biodiversity and resilience.

This study reveals substantial immediate alterations of miRNAs due to experimental environmental conditions, whereas long-term miRNA responses were minor. This indicates a major regulatory role of miRNAs in acute responses to modified environmental conditions. The study adds to the cumulating knowledge of the function of miRNAs and particularly provides data of healthy organisms at different ontogenetic stages.

## Electronic supplementary material

Supplementary tables and figures
