# Supplementary material for: miRNAs regulate acute transcriptional changes in broiler embryos in response to modification of incubation temperature
Source: Sci Rep. 2018 Jul 27;8:11371. doi: 10.1038/s41598-018-29316-7 (PMC6063901; doi:10.1038/s41598-018-29316-7)
Supplement: Supplementary file 1 — Supplementary tables and figures [file 41598_2018_29316_MOESM1_ESM.pdf]

miRNAs regulate acute transcriptional changes in broiler embryos in response to modification of incubation temperature

Watcharapong Naraballobh[1], Nares Trakooljul[1], Eduard Murani[1], Carsten Krischek[2], Sabine Janisch[3], Michael Wicke[3†], Siriluck Ponsuksili[1], Klaus Wimmers[1]*

Supplementary Material

Table S1. Composition of feed stuff for broilers at respective phases

Feeding phase	Starter until day 14	Grower until day 28	Finisher until day 35
Energy (MJ ME/kg)	12.4	12.6	12.7
Crude protein (%)	22	20	18
Methionine (%)	1.33	0.45	0.4
Lysine(%)	1.33	1.15	1.00
Calcium (%)	0.90	0.85	0.80

Table S2. Primers used for quantitative real time PCR

miRNA	Primer sequence
gga-miR-199	GTACAGTAGTCTGCACATTGG
gga-miR-30d	TGTAAACATCCCCGACTGGA
gga-miR-460a	CCTGCATTGTACACACTGTG
gga-miR-100	AACCCGTAGATCCGAACTTGT
gga-miR-133a	TTTGGTCCCCTTCAACCAGCT
gga-miR-222	GAGCTACATCTGGCTACTGG

Table S3. Summary of significant biological functions ($p < 0.05$) of target mRNAs that are related to differentially expressed miRNAs for H10 treatment in embryonic breast and hind muscle tissues

Major category	Ingenuity biofunction	BH *p*-value	Z-score	Top 10 genes	Differentially expressed miRNAs
**Breast H10**					
Cell maintenance, proliferation, differentiation, and replacement	Formation of actin filaments	1.58E-02	–0.728	*ARHGAP18, CDC42BPB, KIRREL, MYH11, NFATC1, PECAM1, PHLDB2, PIK3R1, PPFIA1, ROCK1*	miR-1825, miR-222, miR-739, miR-133, miR-133a-star, miR-17-3p, miR-2970, miR-971, miR-130c, miR-145, miR-363, miR-199a-3p, miR-460-5p, miR-1281, miR-212-star
	Formation of cytoskeleton	1.93E-02	–0.673		
	Interphase	1.09E-02	0.984	*CDKN1B, AKAP12, IL15, PIK3R1, SYK, TCF3, USP8, XRCC5, ADNP, ARFIP2*	miR-222, miR-289, miR-460-5p, miR-199a-3p, miR-212-star, miR-739, miR-578, miR-5109, miR-138, miR-1825, miR-133
	Proliferation of activated T lymphocytes	1.26E-02	1.446		
	Proliferation of thymocytes	3.06E-02	1.715		
	Organization of cytoskeleton	9.72E-03	1.874		
	Organization of cytoplasm	1.47E-02	1.874		
Organismal, organ, and tissue development	Organismal death	8.66E-03	–5.151	*ADNP, BPTF, CUL1, FBN2, HOXA3, JPH1, MYH11, PLXNB2, SLC25A25, TRIM33*	miR-222, miR-289, miR-460-5p, miR-1825, miR-971, let-7, miR-4530, miR-5109, miR-667-star
	Quantity of double-positive thymocyte	2.38E-02	0.128	*CDKN1B, KMT2E, NFATC1, TCF3, TPP2, F3, GUCY1A3, KLF5, SYK, CSPG4*	miR-222, miR-5109, miR-138, miR-3017a, miR-460-5p, miR-289, miR-1825, miR-4530, miR-130c, miR-145, miR-363, miR-133
	Formation of neointima	2.13E-03	0.152		
	Quantity of double-negative T lymphocyte	8.92E-03	0.447		
	Vasculogenesis	3.17E-02	0.882		
	Thermogenesis	2.89E-02	1		
Nutrient metabolism	Synthesis of polysaccharide	1.73E-02	0.447	*CHPF, CHST3, CX3CL1, GALNT5, GBE1, GPAM, PPP1R3B, PPP1R3C, ZFPM2, ADRB2*	miR-199a-3p, miR-212-star, miR-460-5p, miR-5109, miR-133, miR-133a-star, miR-147, miR-2970, miR-1281, miR-667-star, miR-199-star, miR-289
	Metabolism of polysaccharide	1.86E-02	0.816		
	Synthesis of carbohydrate	3.68E-02	1.807		
	Metabolism of phosphatidylinositol	1.89E-02	1.91		

Genetic information and nucleic acid processing	Transcription of DNA	2.11E-02	0.313	*ADRB2, XPO1, ABRA, BACH1, CDKN1B, DCP1A, HOXC8, ILF2, JMJD1C, KDM7A*	miR-5109, miR-199a-3p, miR-212-star, miR-460-5p, miR-289, miR-222, miR-133, miR-3148, miR-93, miR-1825, miR-4530
	Hydrolysis of nucleotide	1.71E-02	1.941		
Cell signaling and interaction	Response of helper T lymphocytes	1.62E-02	–1	*CD276, NFATC1, NFATC2, PIK3R1*	miR-133, miR-130c, miR-145, miR-363, miR-1563, miR-17-3p, miR-2970, miR-425-5p, miR-739
	Hydrolysis of GTP	1.07E-02	1.4	*ADRB2, ARHGAP10, ARHGAP5, RANBP2, XPO1*	miR-199a-3p, miR-212-star, miR-460-5p, miR-222, miR-1825, miR-289, miR-4530
Response to stimuli	Adhesion of monocytes	1.84E-02	0.623	*CX3CL1, F3, IL15, ROCK1*	miR-460-5p, miR-5109, miR-138, miR-212-star
**Hind H10**					
Cell maintenance, proliferation, differentiation, and replacement	Formation of cytoskeleton	0.0275	–1.172	*ARHGAP18, DLC1, MYH11, PIK3R1, PPFIA1, ROCK1, AKAP11, TRAF6, MTM1, ACTA1*	miR-222a-star, miR-1915, miR-199a-5p, miR-4454, miR-4592, miR-638, miR-64b, miR-2861, miR-3960, miR-3885-5p
	Development of cytoplasm	0.0379	–1.172		
	Formation of actin stress fibers	0.0181	–0.849		
	Formation of filaments	0.0065	–0.342		
	Organization of cytoplasm	0.0347	1.219	*AKAP11, ARHGAP5, CELSR1, DLC1, IQGAP1, MTM1, MTUS1, PDZD8, PIK3R1, PLXNB2*	miR-1915, miR-199a-5p, miR-4454, miR-4592, miR-638, miR-64b, miR-1207-5p, miR-22-star, miR-4689, miR-3885-5p, miR-2861, miR-3960
	Organization of cytoskeleton	0.049	1.219		
Organismal, organ, and tissue development	Perinatal death	0.0407	–3.39	*AKAP6, ARHGAP5, ARID4B, HPGD, KMT2E, MAN2A1, MTM1, MYH11, PIK3R1, RAB3C*	miR-212, miR-199a-5p, miR-3885-5p, miR-4592, miR-2861, miR-3960, miR-638, miR-1915, miR-4454
	Size of body	0.00111	3.567	*ACTA1, AKAP6, ARHGAP5, ARID4B, CHD7, CUL4B, GAMT, GPAM, ITSN1, KCNJ16*	miR-222a-star, miR-212, miR-199a-5p, miR-3885-5p, miR-1915, miR-638, miR-2861
Nutrient metabolism	Concentration of phosphatidic acid	0.0069	0.532	*MTM1, FIG4, GPAM, MGLL, NT5E, PIK3R1, PLCB1, PNPLA8*	miR-425-5p, miR-638, miR-199a-5p, miR-212, miR-2861, miR-3960, miR-4592, miR-222
	Hydrolysis of carbohydrate	0.0411	1.972		
Genetic information and nucleic acid processing	Phosphorylation of protein	0.0124	0.896	*N4BP2, AKAP11, AKAP6, ARHGAP5, CCDC88C, CD7, CDKL5, CPNE3, EEF2K, FZD1*	miR-1915, miR-199a-5p, miR-4454, miR-4592, miR-638, miR-212, miR-4689, miR-222, miR-1207-5p, miR-22-star
	Hydrolysis of nucleotide	0.0377	1.067		

Table S4. Summary of significant biological functions ($p < 0.05$) of target mRNAs that are related to differentially expressed miRNAs for L13 treatment in embryonic breast and hind muscle tissues

Major category	Ingenuity biofunction	BH *p*-value	Z-score	Top 10 genes	Differentially expressed miRNAs
**Breast EmL13**					
Cell maintenance, proliferation, differentiation, and replacement	Outgrowth of neurites	0.0131	–1.506	*CD226, CD80, LRRC8A, NOTCH2, PATZ1, PRKCQ, RUNX2, TCF3, PTGS1, SRF*	miR-92a, miR-4900a, miR-344d-2-star, let-7, miR-93, miR-1677, miR-222, miR-4651, miR-762, miR-92b, miR-1908, miR-2436-5p, miR-460-5p, miR-130c, miR-199b, miR-312-5p
	Differentiation of T lymphocytes	0.0215	–0.294		
	Homeostasis of leukocytes	0.0176	–0.156		
	T cell development	0.0214	–0.125		
	Engulfment of leukocytes	0.0029	1.664	*MIF, DNM1, SOAT1, TICAM1, CLIC4, CTNND1, JAM3, PICK1, RNF2, SRF*	miR-5109, miR-2016, let-7, miR-1, miR-17-3p, miR-302a, miR-93, miR-124, miR-1908, miR-222, miR-460-5p, miR-92a, miR-30e, miR-263a-star, miR-1270, miR-199b, miR-4651
	Formation of cells	0.0143	1.709		
	Homing	0.0251	1.947		
	Proliferation of splenocytes	0.0042	2		
	Engulfment of cells	0.0060	2.008		
Organismal, organ, and tissue development	Organismal death	0.0004	–4.096	*AMER1, RUNX2, ARHGAP1, ARID1A, DHCR7, DNM1, INPP5E, PIP5K1C, PTGS1, TCF3*	miR-93, let-7, miR-199b, miR-1, miR-17-3p, miR-302a, miR-1908, miR-2436-5p, miR-460-5p, miR-762, miR-92b, miR-130c, miR-312-5p
	Perinatal death	0.0116	–2.926		
	Neonatal death	0.0077	–2.578		
	Size of bone	0.0117	–1.673		
	Accumulation of leukocytes	0.0235	1.744	*CD80, NOTCH2, PRKCQ, TCF3, MIF, ARHGAP1, HIVEP3, IP6K1, RUNX2, SRF*	miR-93, miR-4900a, let-7, miR-1677, miR-222, miR-4651, miR-762, miR-92a, miR-92b, miR-130c, miR-199b, miR-312-5p
	Quantity of lymphatic system component	0.0196	1.815		
	Development of body trunk	0.0309	1.964		
	Generation of lymphocytes	0.0188	1.987		
	Quantity of double-positive thymocyte	0.0117	2		
Nutrient metabolism	Uptake of D-glucose	0.0064	1.432	*JUP, MIF, NTRK3, PRKAB2,*	miR-5109, let-7, miR-1575, miR-1908, miR-

	Uptake of carbohydrate	0.0109	1.964	*PRKCQ, RAPGEF1, SRF, SLC35B4*	2309, miR-4651, miR-762, miR-1677, miR-222, miR-92a, miR-92b, miR-2413, miR-4900a, miR-1, miR-93, miR-199b
Genetic information and nucleic acid processing	Transcription of DNA	0.0366	0.749	*ARID1A, NOTCH2, RUNX2, SRF, TCF3, CRTC1, PRKCQ, AGO1, ATN1, BCL9*	let-7, miR-93, miR-3128, miR-312-5p, miR-1677, miR-222, miR-4651, miR-762, miR-92a, miR-92b, miR-130c, miR-199b
	Transcription of RNA	0.0327	1.137		
	Expression of RNA	0.0263	1.445		
	Transactivation of RNA	0.0065	1.666		
	Transactivation	0.0004	1.671		
Molecular transport	Transport of protein	0.0049	1.067	*FRAS1, IPO9, JUP, LRP1, NDFIP2, NUP214, XPO5, XPO6, CLIC4, CLNS1A*	miR-5109, miR-312-5p, miR-199-star, miR-199b, miR-2413, miR-31, miR-762, miR-93, let-7, miR-17-3p, miR2275b-5p, miR-1908, miR-4651, miR-30e, miR-263a-star
	Transport of molecule	0.0226	1.21		
Cell signaling and interaction	Adhesion of mononuclear leukocytes	0.0259	0.152	*MIF, PIP5K1C, RALGDS, RAPGEF1, CD80, CLIC4, DNM1, IPO9, JAM3, JUP*	miR-4900a, miR-5109, let-7, miR-1, miR-17-3p, miR-302a, miR-93, miR-124, miR-1908, miR-222, miR-460-5p, miR-92a, miR-2413, miR-4651, miR-762
	Adhesion of connective tissue cells	0.0128	1		
	Phagocytosis by macrophages	0.0131	1		
	Adhesion of immune cells	0.0317	1.299		
Response to stimuli	Migration of phagocytes	0.0102	0.408	*ARHGAP1, JAM3, PRKCQ, SRF, CD80, LRP1, MIF*	miR-93, miR-4900a, miR-124, miR-1908, miR-222, miR-460-5p, miR-5109, miR-92a, let-7, miR-17-3p, miR2275b-5p, miR-1677, miR-4651, miR-762, miR-92b, miR-199b
	Migration of neutrophils	0.0117	1.695		
**Hind EmL13**					
Cell maintenance, proliferation, differentiation, and replacement	Cell death	0.0234	0.915	*ADAMTS20, BLVRA, CTSG, IRS1, MKI67, OSBP2, PCMT1, PRPS1, RCAN2, SRF*	miR-138, miR-222, miR-132, miR-383, miR-271, miR-1245b-3p, miR-2137, miR-3042
	Apoptosis	0.0085	1.269		
Organismal, organ, and tissue development	Growth of connective tissue	0.0090	–1	*IRS1, RAPGEF1, RUNX2, SRF*	miR-271, miR-2137, miR-130c
Response to stimuli	Cell movement of neutrophils	0.0106	1.701	*CTSG, PRKCQ, SRF, TSC1*	miR-138, miR-222, miR-271, miR-383

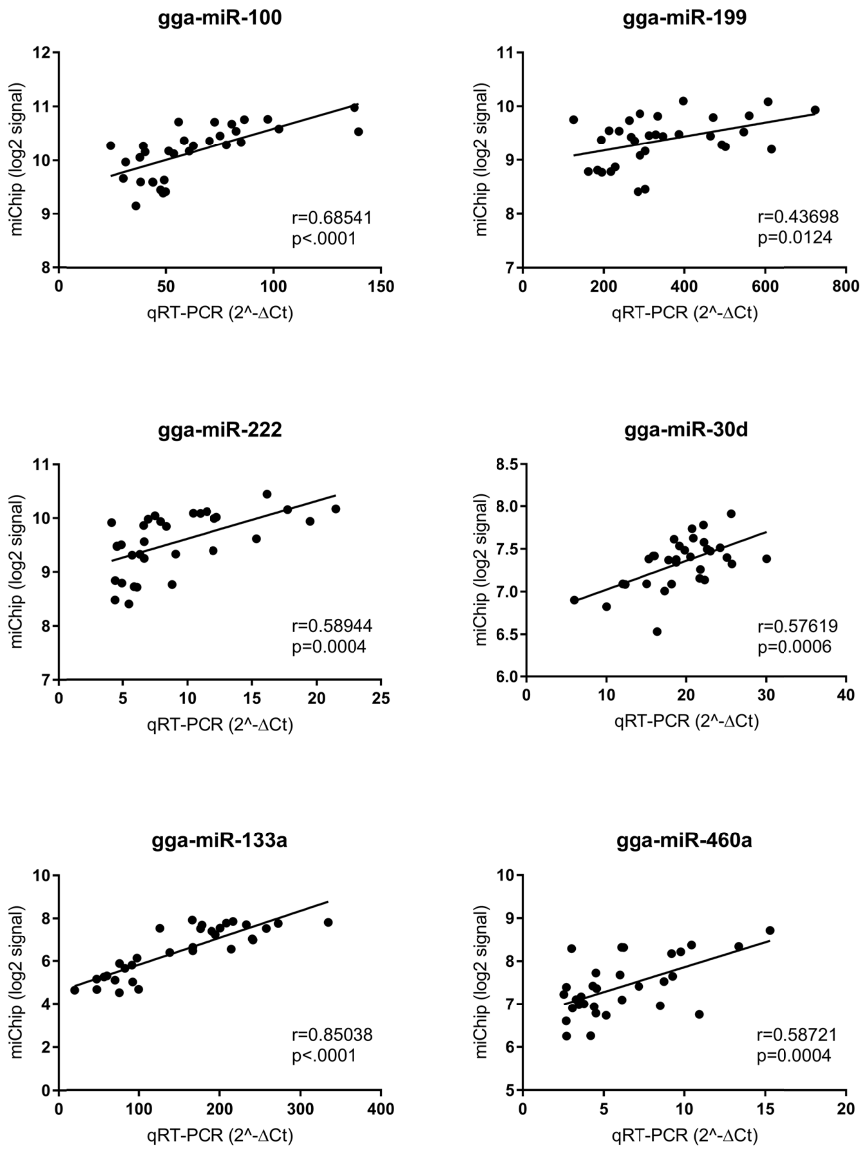

Figure S1. Expression levels of miRNAs obtained from the same samples of embryonic breast muscle (n=32) by microarray and quantitative real time PCR respectively, with Pearson correlation coefficient r and p-values.

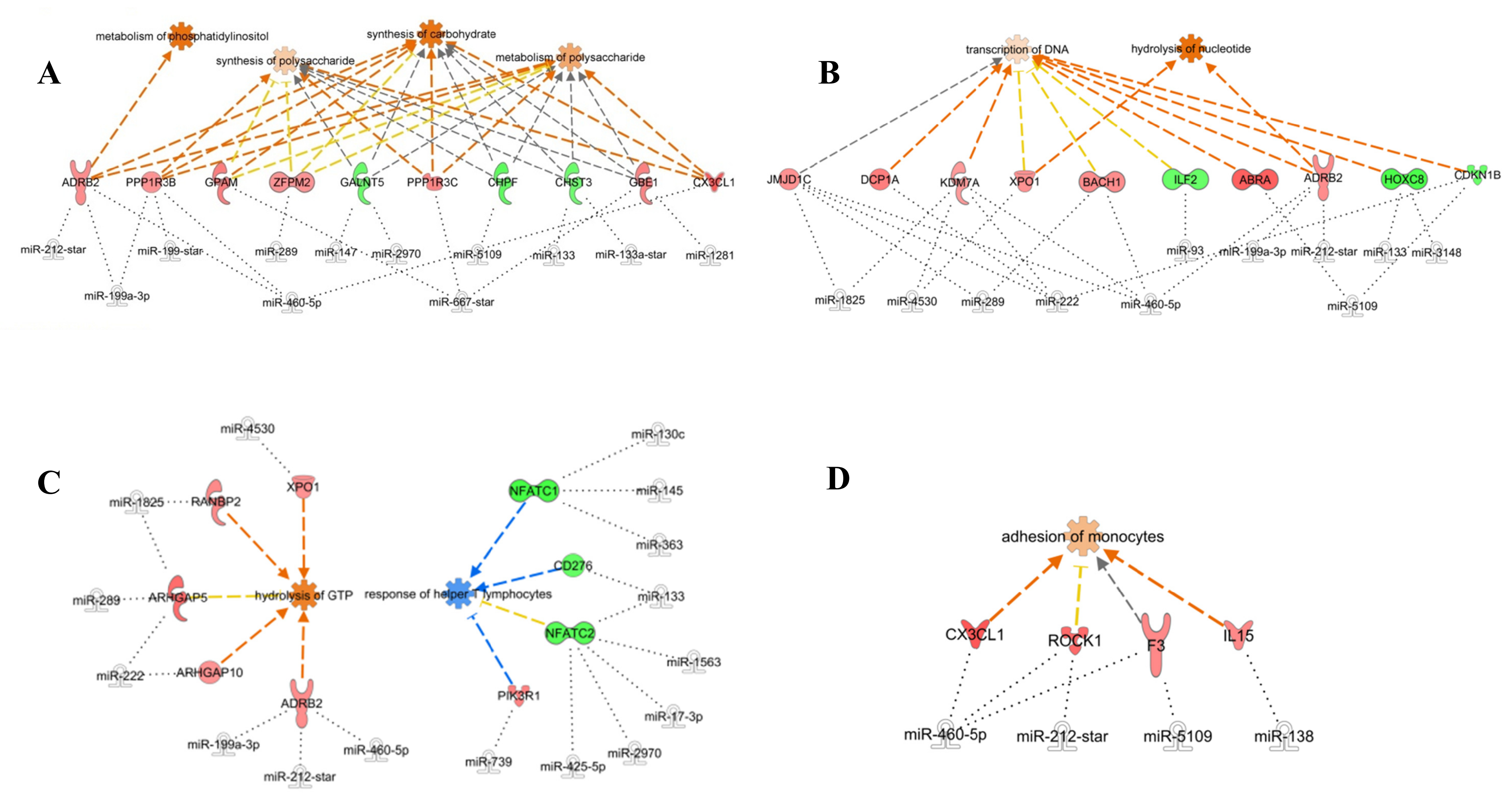

Figure S2. miRNA–mRNA regulatory networks. Representative gene regulatory networks derived from H10 treatment in breast muscle that are related to major functional categories 3 (A), 4 (B), 6 (C), and 8 (D). Activated pathways are orange, deactivated pathways are blue. The networks were generated through the use of IPA (QIAGEN Inc., https://www.qiagenbio-informatics.com/products/ingenuity-pathway-analysis).

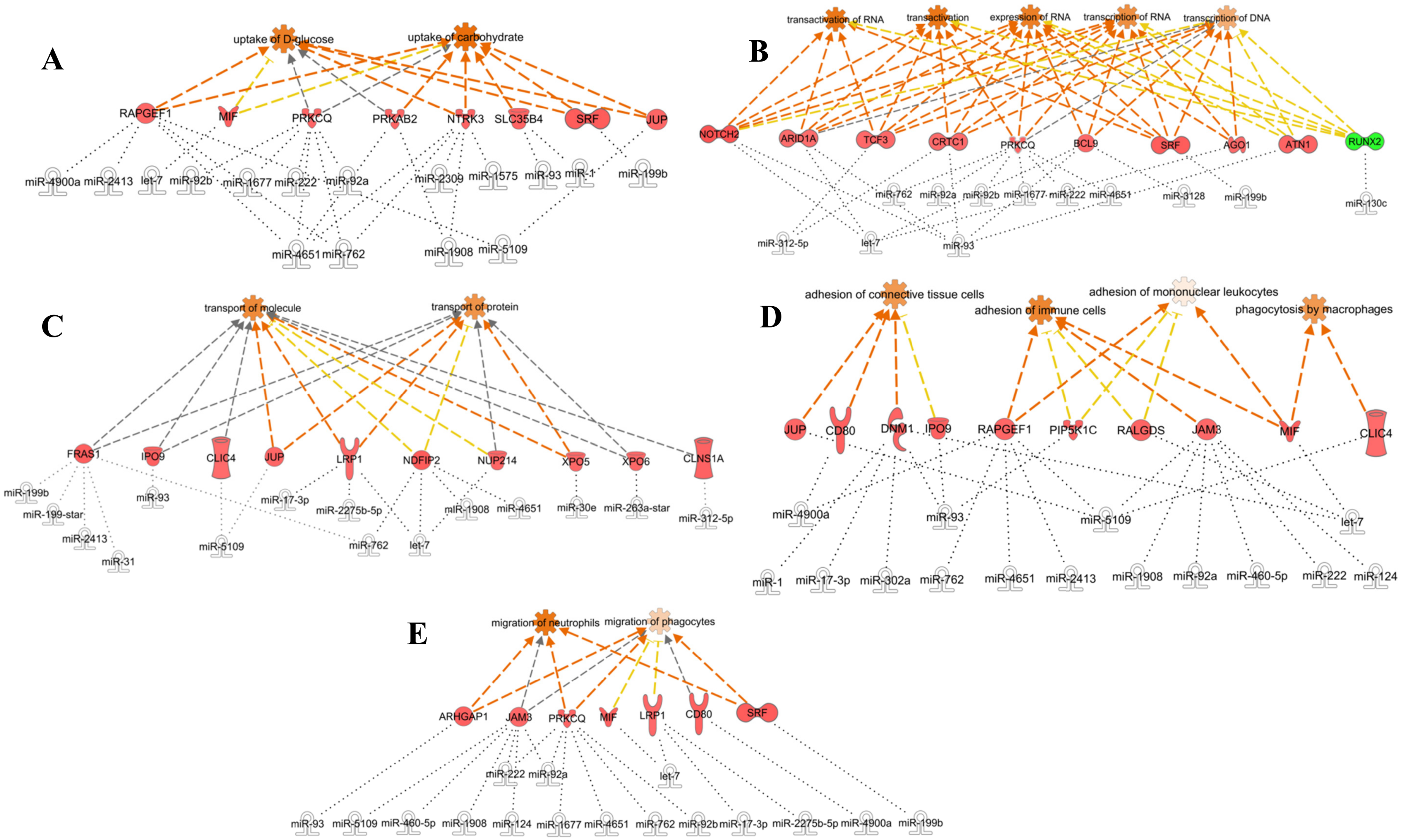

A
uptake of D-glucose
uptake of carbohydrate
RAPGEF1
MIF
PRKCQ
PRKAB2
NTRK3
SLC35B4
SRF
JUP
miR-4900a
miR-2413
let-7
miR-92b
miR-1677
miR-222
miR-92a
miR-2309
miR-1575
miR-93
miR-1
miR-199b
miR-4651
miR-762
miR-1908
miR-5109
B
transactivation of RNA
transactivation
expression of RNA
transcription of RNA
transcription of DNA
NOTCH2
ARID1A
TCF3
CRTC1
PRKCQ
BCL9
SRF
AGO1
ATN1
RUNX2
miR-762
miR-92a
miR-92b
miR-1677
miR-222
miR-4651
miR-3128
miR-199b
miR-130c
miR-312-5p
let-7
miR-93
C
transport of molecule
transport of protein
FRAS1
IPO9
CLIC4
JUP
LRP1
NDFIP2
NUP214
XPO5
XPO6
CLNS1A
miR-199b
miR-199-star
miR-2413
miR-31
miR-93
miR-5109
miR-17-3p
miR-2275b-5p
miR-762
let-7
miR-1908
miR-4651
miR-30e
miR-263a-star
miR-312-5p
D
adhesion of connective tissue cells
adhesion of immune cells
adhesion of mononuclear leukocytes
phagocytosis by macrophages
JUP
CD80
DNM1
IPO9
RAPGEF1
PIP5K1C
RALGDS
JAM3
MIF
CLIC4
miR-4900a
miR-93
miR-5109
let-7
miR-1
miR-17-3p
miR-302a
miR-762
miR-4651
miR-2413
miR-1908
miR-92a
miR-460-5p
miR-222
miR-124
E
migration of neutrophils
migration of phagocytes
ARHGAP1
JAM3
PRKCQ
MIF
LRP1
CD80
SRF
miR-222
miR-92a
let-7
miR-93
miR-5109
miR-460-5p
miR-1908
miR-124
miR-1677
miR-4651
miR-762
miR-92b
miR-17-3p
miR-2275b-5p
miR-4900a
miR-199b

Figure S3. miRNA–mRNA regulatory networks. Representative gene regulatory networks derived from L13 treatment in breast muscle that are related to major functional categories 3 (A), 4 (B), 5 (C), 6 (D), and 8 (E). Activated pathways are orange. The networks were generated through the use of IPA (QIAGEN Inc., https://www.qiagenbio-informatics.com/products/ingenuity-pathway-analysis).

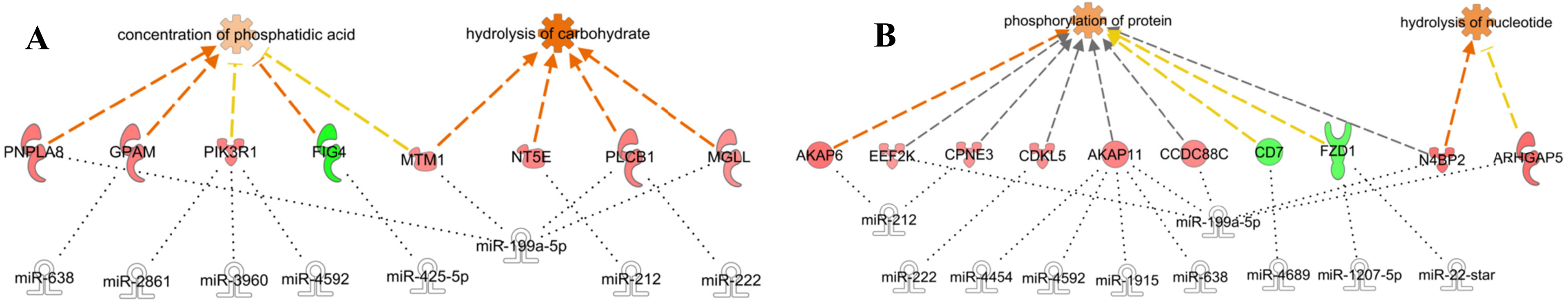

Figure S4. miRNA–mRNA regulatory networks. Representative gene regulatory networks derived from H10 treatment in hind muscle that are related to major functional categories 3 (A) and 4 (B). Activated pathways are orange, while deactivated pathways are blue. The networks were generated through the use of IPA (QIAGEN Inc., https://www.qiagenbio-informatics.com/products/ingenuity-pathway-analysis).

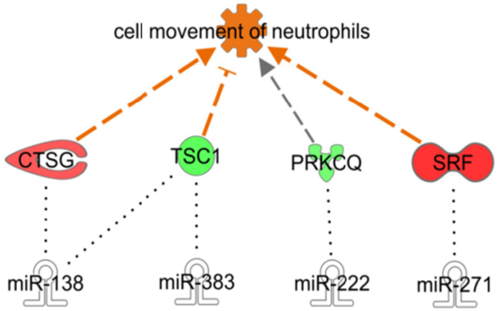

Figure S5. miRNA-mRNA regulatory networks. Representative gene regulatory networks derived from H10 treatment in hind muscle that are related to major functional category 8. Activated pathways are orange. The networks were generated through the use of IPA (QIAGEN Inc., https://www.qiagenbio-informatics.com/products/ingenuity-pathway-analysis).
